# Integrative Transcriptomics and Machine Learning Identify Temporal Stress Signatures and Astrocytic Neurocan Associated With Hypothalamic Injury

**DOI:** 10.1002/cns.71219

**Published:** 2026-10-23

**Authors:** Weihao Zhu, Chenyu Li, Mengting He, Rui Shi, Xia Liu, Weibo Shi, Bin Cong

**Affiliations:** ^1^ Hebei Medical University Postdoctoral Research Station in Basic Medicine Shijiazhuang China; ^2^ Hebei Key Laboratory of Forensic Medicine, Collaborative Innovation Center of Forensic Medical Molecular Identification, College of Forensic Medicine Hebei Medical University Shijiazhuang China; ^3^ Shenzhen Second People's Hospital The First Affiliated Hospital of Shenzhen University Shenzhen China

**Keywords:** hypothalamus, machine learning, Ncan, stress, transcriptome

## Abstract

**Background:**

Stress‐related neurological dysfunction evolves dynamically, but molecular markers distinguishing stress stages remain limited. We aimed to characterize temporal hypothalamic transcriptomic changes and identify molecular signatures for stress‐stage discrimination.

**Methods:**

Whole‐transcriptome sequencing was performed in mouse hypothalami after stress. Differential expression, temporal clustering, co‐expression network analysis, and machine learning were integrated to identify stress‐associated mRNA and miRNA signatures. Candidate markers were validated by RT‐qPCR and single‐nucleus RNA sequencing. Ncan function was further evaluated by protein supplementation, perineuronal net analysis, behavioral testing, and regulatory network analysis.

**Results:**

mRNAs exhibited strong temporal sensitivity and captured stage‐specific transcriptional remodeling, whereas miRNAs primarily reflected stress regardless of duration. A six‐mRNA signature (Ncan, Mt2, Nab2, Ptch1, and Catspere1/Catspere2) accurately distinguished control, acute stress, and chronic stress (accuracy, 1.000), while an 11‐miRNA signature robustly classified stress states (accuracy, 0.944). Both signatures were validated by RT‐qPCR and nested cross‐validation, with accuracies of 0.898 and 0.890, respectively. Ncan was consistently top‐ranked, predominantly expressed in astrocytes, progressively downregulated by stress, and its supplementation restored PNNs and alleviated depressive‐like behaviors. Virtual Ncan knockout disrupted the stress network, with Ctcf predicted as its upstream regulator.

**Conclusion:**

Our study identifies distinct stress‐stage and stress‐state transcriptomic signatures and highlights astrocytic Ncan as a candidate biomarker that may contribute to stress‐induced neural injury by regulating PNN homeostasis.

## Graphical abstract

Our research journey revealed a clear split: mRNAs track stress stages, while miRNAs sense stress states. We built a validated predictive model from just 6 markers. The star was Ncan—an astrocytic proteoglycan—which restored neuronal nets and eased depression; virtual knockout confirmed its central role in stress networks.

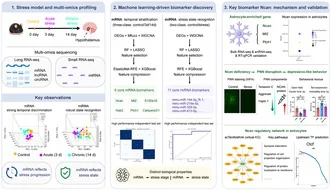

## 1 Introduction

Stress is an adaptive response that enables organisms to cope with environmental challenges; however, prolonged stress disrupts hypothalamic–pituitary–adrenal (HPA) axis function, induces neuroinflammation and metabolic dysfunction, and contributes to the development of depression, anxiety, and neurodegenerative diseases [1, 2, 3, 4, 5]. Accurate discrimination of stress stages is critical for early intervention, yet current assessments rely largely on behavioral testing and endocrine markers such as cortisol and CRH, which are susceptible to circadian fluctuations and provide limited information on brain‐specific pathological changes [6, 7]. As the central regulator of the HPA axis, the hypothalamus orchestrates stress responses, but its dynamic molecular remodeling during stress progression remains poorly understood [8, 9, 10, 11].

Advances in transcriptomic sequencing have enabled systematic identification of stress‐associated molecular alterations. mRNAs directly reflect transcriptional activity, whereas miRNAs are stable post‐transcriptional regulators, suggesting complementary value for characterizing stress adaptation [12, 13]. However, the distinct contributions of these transcriptomic layers across stress progression remain unclear. Moreover, conventional differential expression analyses are limited by high‐dimensional data and poor generalizability, whereas machine‐learning approaches have rarely been applied to temporal stress stratification or combined with experimental validation [13, 14, 15, 16].

Among the candidate biomarkers identified, neurocan (Ncan), a major component of perineuronal nets (PNNs), consistently emerged as the most robust feature across independent machine‐learning models. Ncan has been implicated in synaptic plasticity, neural circuit stability, and psychiatric disorders [17, 18, 19]. Although chronic stress has been associated with PNN disruption [20, 21], the cellular origin, regulatory mechanisms, and functional significance of Ncan in hypothalamic stress responses remain unknown.

Here, we integrated whole‐transcriptome sequencing, single‐nucleus RNA sequencing (snRNA‐seq), and machine‐learning analyses to characterize hypothalamic molecular remodeling during acute and chronic stress. We established mRNA‐ and miRNA‐based classifiers to compare their abilities to distinguish stress duration and stress state. Guided by machine‐learning prioritization, we further combined single‐cell localization, regulatory network analysis, in silico perturbation, and functional experiments to investigate the cellular source, regulatory mechanisms, and biological role of Ncan. Our findings define complementary functions of mRNAs and miRNAs in stress adaptation, establish a framework for temporal stress stratification, and identify astrocyte‐derived Ncan as a potential regulator of PNN homeostasis during stress‐induced hypothalamic injury.

## 2 Materials and Methods

### 2.1 Animals and Stress Paradigm

Given that sex differences can influence stress responses in mice [22, 23], this study utilized male C57BL/6N mice (7–8 weeks old, weighing 22 ± 2 g) obtained from Beijing Vital River Laboratory Animal Technology Co. Ltd. Mice were housed under SPF conditions (22°C ± 2°C, 50%–60% humidity, 12 h light/dark cycle) with free access to food and water. After 1 week of acclimation, mice were randomly assigned to three groups: control, acute, and chronic stress. Randomization was performed using a computer‐generated random number sequence. All behavioral tests and sample collections were conducted by investigators blinded to group assignment. Exclusion criteria were predefined as follows: mice exhibiting > 20% body weight loss, signs of severe illness, or failure to complete the full stress paradigm were excluded from subsequent analyses. Acute and chronic stress were induced by restraint stress (6 h/day) for 3 or 14 consecutive days, respectively. Control mice were handled without restraint. Following the final stress session, hypothalamic tissues were collected and stored at −80°C.

### 2.2 Whole‐Transcriptome Sequencing of the Hypothalamus

For whole‐transcriptome sequencing, hypothalamic tissues from 15 mice per group (control, acute and chronic stress) were used, totaling 45 samples. Total RNA was extracted using TRIzol (Invitrogen, USA), and RNA samples with an RNA integrity number (RIN) ≥ 7.0 were used for library preparation. Long‐RNA libraries were generated following rRNA depletion and sequenced as paired‐end 150‐bp reads (2 × 150 bp) on an Illumina NovaSeq 6000 platform. Small RNA libraries were prepared using the TruSeq Small RNA Sample Preparation Kit and sequenced as single‐end 50‐bp reads (1 × 50 bp) on an Illumina HiSeq 2000/2500 platform by LC‐Bio Technologies (Hangzhou, China).

Sequencing quality was assessed based on standard quality‐control metrics, including sequencing depth, base quality, Q30, GC content, and read quality. Low‐quality reads and adapter sequences were removed before downstream analysis. The resulting high‐quality reads were aligned to the mouse reference genome, and gene expression was quantified using standard transcriptomic pipelines. Long non‐coding RNAs and circular RNAs were identified according to established annotation and prediction procedures, and miRNAs were annotated based on miRBase. Expression data were normalized before downstream differential expression and functional enrichment analyses. Each mouse was treated as an independent biological replicate throughout the whole‐transcriptome analysis.

### 2.3 Principal Component Analysis (PCA) and Partial Least Squares‐Discriminant Analysis (PLS‐DA)

PCA and PLS‐DA were performed on normalized mRNA, lncRNA, circRNA, and miRNA expression matrices to evaluate sample clustering and identify potential outliers. Analyses were conducted in R using the stats, scatterplot3d, metaX, and ggplot2 packages based on normalized expression matrices.

### 2.4 Differential Expression and Functional Enrichment Analyses

Differential expression analyses were performed using DESeq2. Differentially expressed genes (DEGs) and differentially expressed miRNAs (DEMs) were defined as |log_2_FC| > 1 and *p* < 0.05. GO and KEGG enrichment analyses were conducted using clusterProfiler, with adjusted *p* < 0.05 considered significant.

### 2.5 Mfuzz Temporal Clustering Analysis

Mfuzz (R package v2.54.0) was used to identify genes and miRNAs displaying continuous temporal expression patterns during stress progression. Candidate molecules were obtained by intersecting temporally regulated clusters with DEGs or DEMs.

### 2.6 Weighted Gene Co‐Expression Network Analysis (WGCNA)

WGCNA was performed to identify mRNA and miRNA modules associated with stress progression. Module eigengenes were correlated with stress phenotypes to identify modules significantly associated with stress progression. Functionally relevant modules were subjected to GO and KEGG enrichment analyses.

### 2.7 Machine Learning Feature Selection and Model Construction

Candidate features were obtained by integrating differential expression analysis, Mfuzz clustering, and WGCNA. Samples were randomly divided into training (70%) and testing (30%) sets, with feature selection and model development performed exclusively on the training set, while the test set was reserved solely for model evaluation. Feature selection was performed using RF and LASSO regression, followed by ElasticNet‐based recursive feature elimination (RFE) and XGBoost optimization to reduce feature redundancy. Multiple machine‐learning models, including Ridge, LASSO, ElasticNet, PLS‐DA, linear SVM, RF, RBF‐SVM, XGBoost, and LightGBM, were constructed using the selected features. Model performance was evaluated by repeated cross‐validation, independent testing, leave‐one‐out cross‐validation (LOOCV), and SHAP analysis using Accuracy, Balanced Accuracy, Macro‐F1, and ROC‐AUC.

### 2.8 RT‐qPCR Validation

RT‐qPCR was performed using SYBR Green chemistry. Relative expression levels were calculated using the 2^−ΔΔCt^ method with Gapdh and U6 as internal controls.

U6: F‐CTCGCTTCGGCAGCACA, R‐AACGCTTCACGAATTTGCGT;

miRNA: R‐AGTGCAGGGTCCGAGGTATT;

mmu‐miR‐144‐3p_R‐1: F‐GCGGGCTACAGTATAGATGATGTAC;

mmu‐miR‐219a‐5p: F‐GGTGATTGTCCAAACGCAATTCT;

mmu‐miR‐329‐3p: F‐GGAACACACCCAGCTAACCTT;

mmu‐miR‐672‐5p: F‐TGAGGTTGGTGTACTGTGTGTG;

pal‐miR‐9226‐5p L‐1 1ss4AG: F‐AGTTTCAGGTCCCTGTTCGG;

mmu‐miR‐760‐3p_R + 2: F‐CTCTGGGTCTGTGGGGAGT;

Gapdh: F‐GGCTCATGACCACAGTCCAT, R‐ACATTGGGGGTAGGAACACG;

Ptch1: F‐CGTTTCTTGCTCTTGGTGTTG, R‐AGTCCTGTCCTCAAATGGAATC;

S100a16: F‐AAAGATGCTCCAGAGAGAGTTG, R‐TCCAGTATTCGTCAAAGCAGAT;

Mt2: F‐ACCGATCTCTCGTCGATCTT, R‐TGCAGGAAGTACATTTGCATTG;

Catspere2: F‐GGCTGGAATTCACACGATAGA, R‐CAACTGTGAAATACCACAAGTAGC;

Catspere1: F‐CAGGAACATCCGCATAGTGATA, R‐TCAGGCATGGCAGAGTAAAG;

Nab2: F‐GCATCCCTGTTGAAGCTGAATA, R‐CTGTACTTCCGGATCTCCTCTT;

Machine‐learning models were constructed using RT‐qPCR data within a nested cross‐validation framework. Ridge, LASSO, ElasticNet, linear SVM, RBF‐SVM, PLS‐DA, Shrinkage LDA, ExtraTrees, and Gaussian Naive Bayes were evaluated. Model performance was optimized using Macro‐F1, and SHAP analysis was performed to assess feature importance.

### 2.9 snRNA‐Seq and Data Analysis

For snRNA‐seq, hypothalamic tissues from 3 mice per group (control‐3d, stress‐3d, control‐14d, and stress‐14d) were used, totaling 12 samples. snRNA‐seq libraries were generated using the Chromium Single Cell platform (10× Genomics) and sequenced on the Illumina NovaSeq 6000 platform by Lianchuan Biotechnology Co. Ltd. (Hangzhou, China). Raw sequencing data were processed using Cell Ranger to generate gene–nucleus expression matrices. Quality control and downstream analyses were performed using Seurat. Nuclei with low gene counts, low transcript counts, or a high proportion of mitochondrial transcripts were excluded according to predefined quality‐control criteria. Potential doublets and low‐quality nuclei were also removed before downstream analysis. The retained expression matrix was normalized using the SCTransform normalization method, followed by highly variable gene selection, dimensionality reduction, clustering, and cell‐type annotation. Cell identities were assigned based on established marker genes and a published hypothalamic cell atlas [24].

To avoid pseudoreplication, nuclei were aggregated by mouse and cell type to generate animal‐level pseudobulk profiles, with each mouse treated as an independent biological replicate for differential expression analysis. Batch effects were assessed using sample‐level PCA and clustering, and corrected before dimensionality reduction and clustering where applicable. Differential expression analysis was performed using animal‐level pseudobulk data.

The expression of the seven machine‐learning–identified mRNAs was examined across major hypothalamic cell types, with Ncan, Mt2, and Ptch1 further analyzed in astrocytes and their subpopulations. GO enrichment of Ncan‐associated genes was performed using clusterProfiler with the org.Mm.eg.db annotation database.

### 2.10 FITC‐Conjugated *Wisteria floribunda* Agglutinin (FITC‐WFA) Staining

FITC‐conjugated *Wisteria floribunda* agglutinin staining (20 μm thick frozen section) was performed to evaluate hypothalamic PNN integrity. Fluorescence images were acquired under identical settings, and PNN intensity was quantified using ImageJ.

### 2.11 Intracerebroventricular Administration of Recombinant Ncan Protein

Recombinant mouse Ncan protein (3 μg in 5 μL sterile saline) was injected into the lateral ventricle (AP −0.82 mm, ML −1.49 mm, DV −1.97 mm from bregma) of anesthetized mice via a Hamilton syringe at a rate of 0.5 μL/min. The needle was left in place for 5 min before slow withdrawal. Control mice received either an equal volume of vehicle (sterile saline) or heat‐inactivated Ncan protein. Following a 7‐day recovery period, mice underwent stress exposure and behavioral testing, with daily health monitoring throughout.

### 2.12 Behavioral Tests

Anxiety‐ and depressive‐like behaviors were evaluated using the open field test (OFT) and tail suspension test (TST). For the OFT, mice were allowed to explore a 40 × 40 × 40 cm arena for 5 min, and center time and total travel distance were analyzed using the EthoVision XT tracking system. For the TST, mice were suspended by the tail for 6 min, and immobility time was recorded as an index of depressive‐like behavior.

### 2.13 Western Blotting

Total protein was extracted from hypothalamic tissues using RIPA lysis buffer containing protease and phosphatase inhibitors. Equal amounts of protein were separated by SDS–PAGE, transferred onto PVDF membranes, and incubated with primary antibodies against Tenascin C (1:400), Aggrecan (1:1000), Hapln1 (1:500), GAPDH (1:100,000), and Tubulin (1:100,000). Protein signals were detected using an Odyssey infrared imaging system (LI‐COR Biosciences, USA), quantified with ImageJ, and normalized to GAPDH or Tubulin.

### 2.14 Virtual Knockout Analysis of Ncan

Virtual gene knockout analysis was performed using scTenifoldKnk based on astrocyte snRNA‐seq data to evaluate transcriptomic perturbations following Ncan deletion. Differentially perturbed genes were subjected to intersection and functional enrichment analyses using Metascape to identify stress‐associated biological processes regulated by the Ncan network.

### 2.15 Transcription Factor (TF) Regulatory Network Analysis in Astrocytes

TF regulatory networks were inferred using the pySCENIC workflow. Candidate upstream regulators of Ncan were identified based on regulon activity and expression patterns. TF binding motifs within the Ncan promoter were further predicted using the JASPAR database, and binding sites with relative scores > 0.9 were considered high‐confidence.

### 2.16 Statistical Analysis

Statistical analyses were performed using R (v4.3.0) and Python (v3.10). Data are presented as mean ± SEM. Appropriate parametric or nonparametric tests were applied as required. A two‐sided *p* < 0.05 was considered statistically significant.

## 3 Results

### 3.1 Distinct Transcriptomic Signatures of mRNAs and miRNAs Distinguish Stress Duration and Stress States

Whole‐transcriptome sequencing generated high‐quality long‐RNA and small‐RNA datasets suitable for downstream analyses (Figure S1A,B). PCA identified one control sample (Control‐13) as an outlier in both datasets, which was excluded from subsequent analyses (Figure S1C,D). To compare the ability of different RNA classes to distinguish stress conditions, PCA and PLS‐DA were performed using mRNA, lncRNA, circRNA, and miRNA expression profiles. mRNAs showed the clearest separation among the control, 3‐day stress, and 14‐day stress groups, whereas miRNAs distinguished stress conditions with moderate separation. In contrast, lncRNAs and circRNAs exhibited substantial overlap between groups, indicating limited discriminatory ability (Figure S2A). Consistently, PLS‐DA confirmed that mRNAs and miRNAs achieved markedly better classification performance than lncRNAs and circRNAs (Figure S2B,C). These results demonstrate distinct transcriptomic responses among RNA classes during stress progression, with mRNAs providing superior resolution of stress duration and miRNAs showing greater ability to distinguish stress states. Therefore, subsequent analyses focused on mRNAs and miRNAs.

### 3.2 Temporal Divergence of mRNA and miRNA Regulatory Programs During Acute and Chronic Stress

To characterize transcriptomic dynamics during stress progression, differential expression analyses were performed in the 3‐day and 14‐day stress groups. A total of 376 and 416 differentially expressed genes (DEGs) were identified at 3 and 14 days, respectively (Figure 1A,B). KEGG analysis revealed persistent enrichment of inflammatory and metabolic pathways, including PPAR signaling and cytokine–cytokine receptor interaction, across both stages. However, stage‐specific differences were evident: early stress was predominantly associated with lipid metabolism and inflammatory pathways, whereas prolonged stress preferentially involved steroid hormone biosynthesis and retinol metabolism, indicating progressive neuroendocrine dysregulation (Figure 1A,B).

**FIGURE 1:**
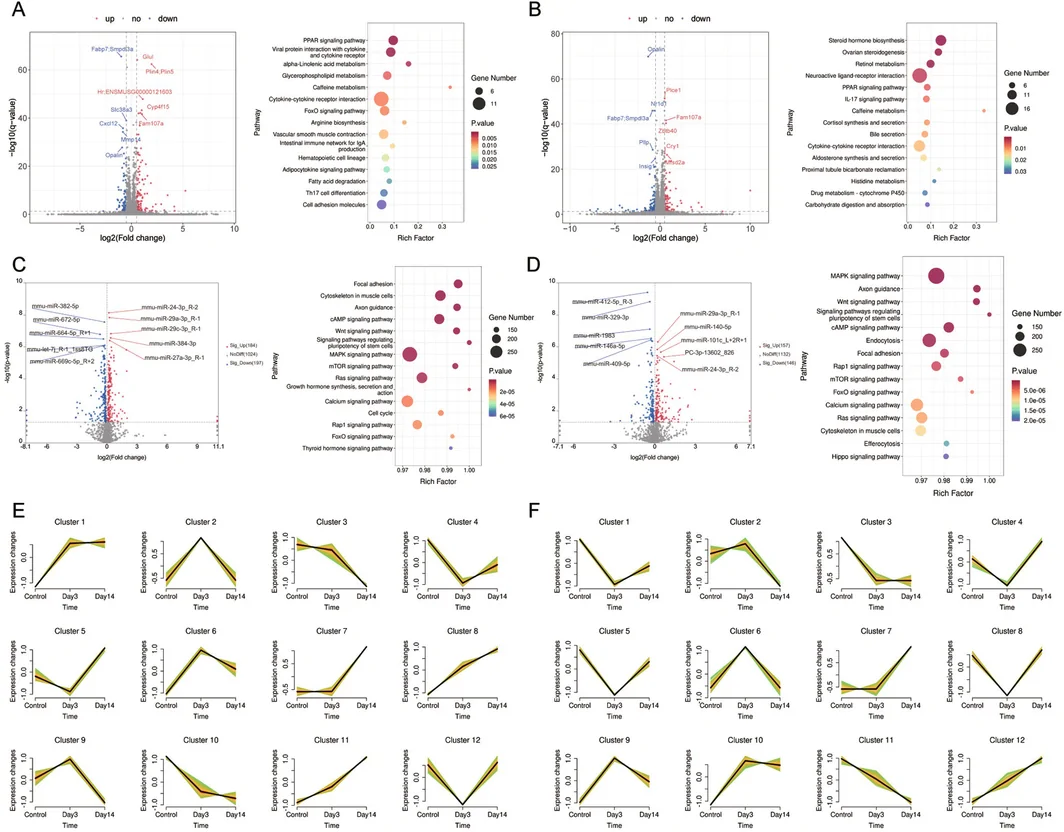
Dynamic regulatory patterns of mRNAs and miRNAs during acute and chronic stress. (A–D) Differential expression analysis (left) and KEGG pathway enrichment (right) based on mRNA and miRNA expression profiles in the 3‐day and 14‐day stress groups. (A) mRNA, 3‐day stress group. (B) mRNA, 14‐day stress group. (C) miRNA, 3‐day stress group. (D) miRNA, 14‐day stress group. (E, F) Mfuzz temporal clustering analysis based on mRNA and miRNA expression profiles. (E) mRNA. (F) miRNA.

In contrast, miRNA expression was more conserved across stress stages, with 381 and 303 differentially expressed miRNAs (DEMs) identified after 3 and 14 days of stress, respectively (Figure 1C,D). Most enriched pathways were shared between stages and were mainly related to neuronal signaling, including axon guidance, MAPK signaling, and Wnt signaling. Only a limited number of pathways showed stage‐specific enrichment (Figure 1C,D). These results indicate that mRNAs exhibit greater temporal plasticity during stress progression, whereas miRNAs maintain relatively stable regulatory programs, supporting their respective roles in distinguishing stress duration and stress state.

### 3.3 Temporal Co‐Expression Network Analysis Identifies Stress Progression–Associated Transcriptional Modules

To identify transcriptomic features associated with stress progression, Mfuzz clustering was performed on mRNA and miRNA expression profiles. Two mRNA clusters (Cluster 8 [*n* = 2159] and Cluster 11 [*n* = 2291]) displayed sustained upregulation across stress progression, and their intersection with differentially expressed genes identified 11 core genes with stable temporal expression patterns (Figure 1E; Figure S3A,B). In parallel, two miRNA clusters (Cluster 11 [*n* = 112] and Cluster 12 [*n* = 100]) exhibited persistent upregulation or downregulation, yielding 16 core differentially expressed miRNAs with distinct temporal dynamics (Figure 1F; Figure S3C,D).

Subsequently, WGCNA was performed to identify coordinated transcriptomic modules associated with stress progression. For mRNAs, 32 co‐expression modules were identified (*β* = 3, *R* ^2^ = 0.850), among which the MEbrown (3d: *r* = −0.102, *p* = 0.509; 14d: *r* = −0.355, *p* = 0.018) and MElightcyan (3d: *r* = −0.131, *p* = 0.395; 14d: *r* = −0.472, *p* = 0.001) modules showed progressive decreases during stress progression and were positively correlated with the control group (Figure S4A–C). GO analysis indicated that these modules were primarily enriched in metabolic processes, including ethanol and linoleic acid metabolism as well as endocannabinoid signaling, suggesting pronounced metabolic reprogramming during stress progression (Figure S4D,E). For miRNAs, 10 co‐expression modules were identified (*β* = 2, *R* ^2^ = 0.850), with the MEbrown (3d: *r* = 0.185, *p* = 0.230; 14d: *r* = −0.489, *p* = 0.0008) and MEblue (3d: *r* = 0.072, *p* = 0.644; 14d: *r* = 0.269, *p* = 0.077) modules exhibiting opposite temporal expression patterns across stress progression (Figure S4F–H). Functional enrichment demonstrated that these modules were mainly associated with transcriptional regulation, phosphorylation, and signal transduction (Figure S4I,J), consistent with the regulatory roles of miRNAs during stress progression.

### 3.4 Machine‐Learning Integration Identifies a Compact mRNA Signature for Robust Stress‐Stage Classification

To integrate transcriptomic features identified by differential expression analysis, Mfuzz clustering, and WGCNA, RF and LASSO regression were applied for feature selection. RF retained 296 candidate genes, whereas LASSO identified 26 genes. Integration of both approaches yielded 304 candidate genes for downstream modeling (Figure 2A–C; Table S1). Functional enrichment analysis indicated that these genes were mainly associated with intracellular signaling, cell adhesion, and brain development (Figure 2D).

**FIGURE 2:**
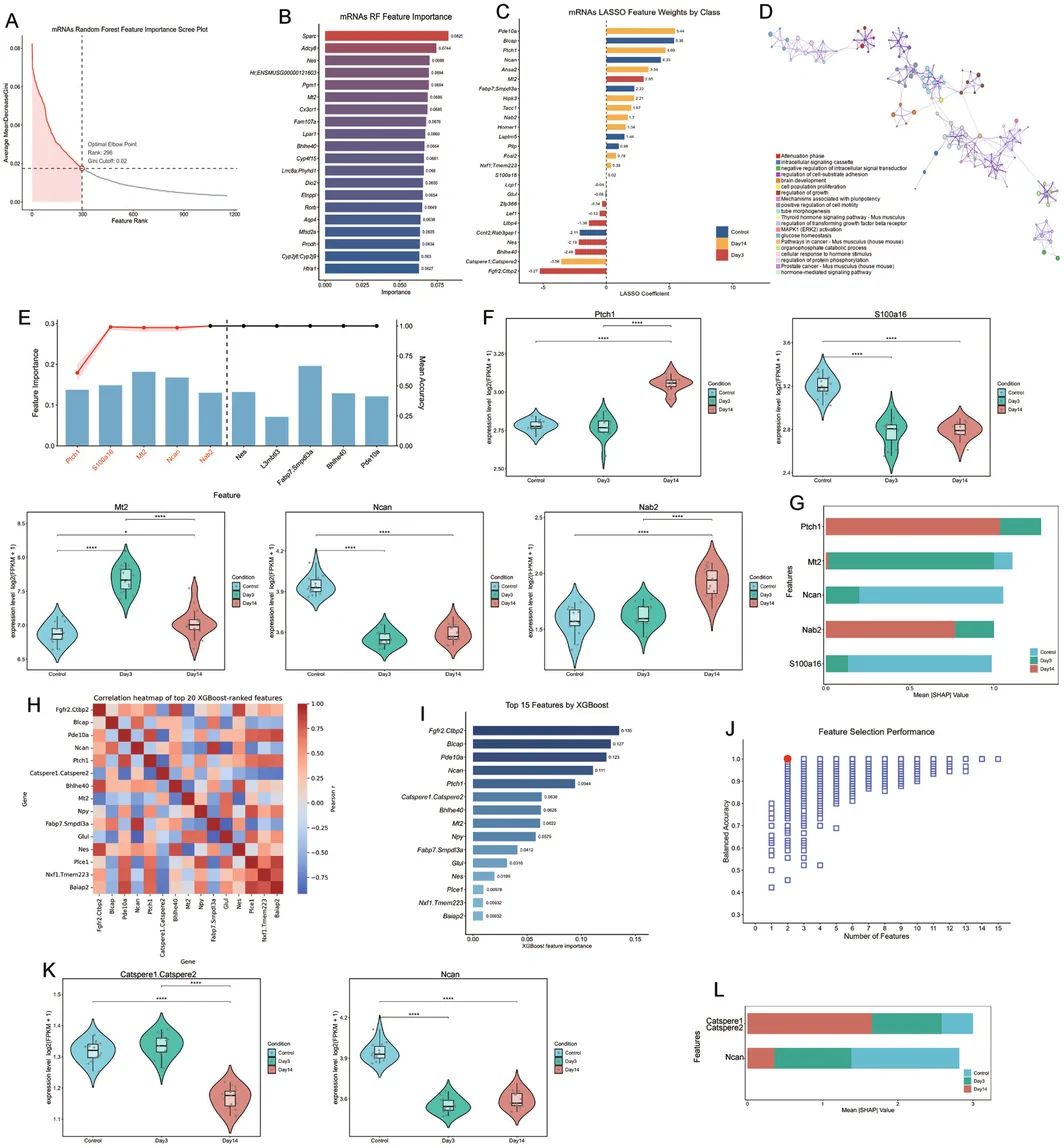
Multi‐layer machine learning identifies the optimal mRNA signature for stress stratification. (A) Scree plot of feature importance derived from the RF model, with the mathematical elbow point identified using the Kneedle algorithm. (B) Top 20 genes ranked by RF importance. (C) Twenty‐six genes selected by multinomial LASSO regression. (D) Functional enrichment network of the 304 integrated genes used for model construction. (E) Feature reduction of the 304 genes using the ElasticNet‐RFE strategy, showing optimal model performance with the top five features. (F) Violin plots showing the expression levels of Ncan, Mt2, Nab2, Ptch1, and S100a16. (G) SHAP summary plots of Ncan, Mt2, Nab2, Ptch1, and S100a16. (H) Pearson correlation analysis of the top 15 features ranked by XGBoost. (I) Top 15 feature genes selected by XGBoost. (J) Exhaustive subset search based on the top 15 features, followed by five‐fold stratified cross‐validation to identify the optimal feature combination. (K), Violin plots showing the expression levels of Ncan and Catspere2/Catspere1. (L) SHAP summary plots of Ncan and Catspere2/Catspere1.

Nine machine‐learning algorithms were subsequently evaluated to construct a three‐class classifier for stress‐stage stratification. Most models achieved excellent classification performance (Figure S5A–I). Among them, Ridge regression, LASSO, ElasticNet, and linear SVM showed the most robust and consistent performance across cross‐validation and independent testing, whereas tree‐based models, including RF, XGBoost, and LightGBM, exhibited greater variability despite maintaining high predictive accuracy (Table S2).

To further reduce model complexity, ElasticNet‐RFE and XGBoost‐based feature ranking were performed independently. ElasticNet‐RFE identified an optimal five‐gene signature comprising Ncan, Mt2, Nab2, Ptch1, and S100a16, whereas XGBoost prioritized complementary feature combinations. Integration of both strategies generated a compact six‐gene signature for downstream analyses (Figure 2E–L). PCA (*r* = 0.752, *p* = 0.001) and PLS‐DA demonstrated that the six‐gene signature clearly distinguished the control, 3‐day stress, and 14‐day stress groups (Figure 3A–C). Classification models reconstructed using these six genes maintained excellent performance across nearly all algorithms, confirming that substantial feature reduction did not compromise predictive accuracy (Figure 3D–G; Table S3). Leave‐one‐out cross‐validation further supported the robustness and generalizability of the classifier. SHAP analysis consistently identified Mt2, Ncan, and Ptch1 as the major contributors to model prediction (Figure 3H), suggesting that these genes represent the principal molecular features underlying temporal stress stratification.

**FIGURE 3:**
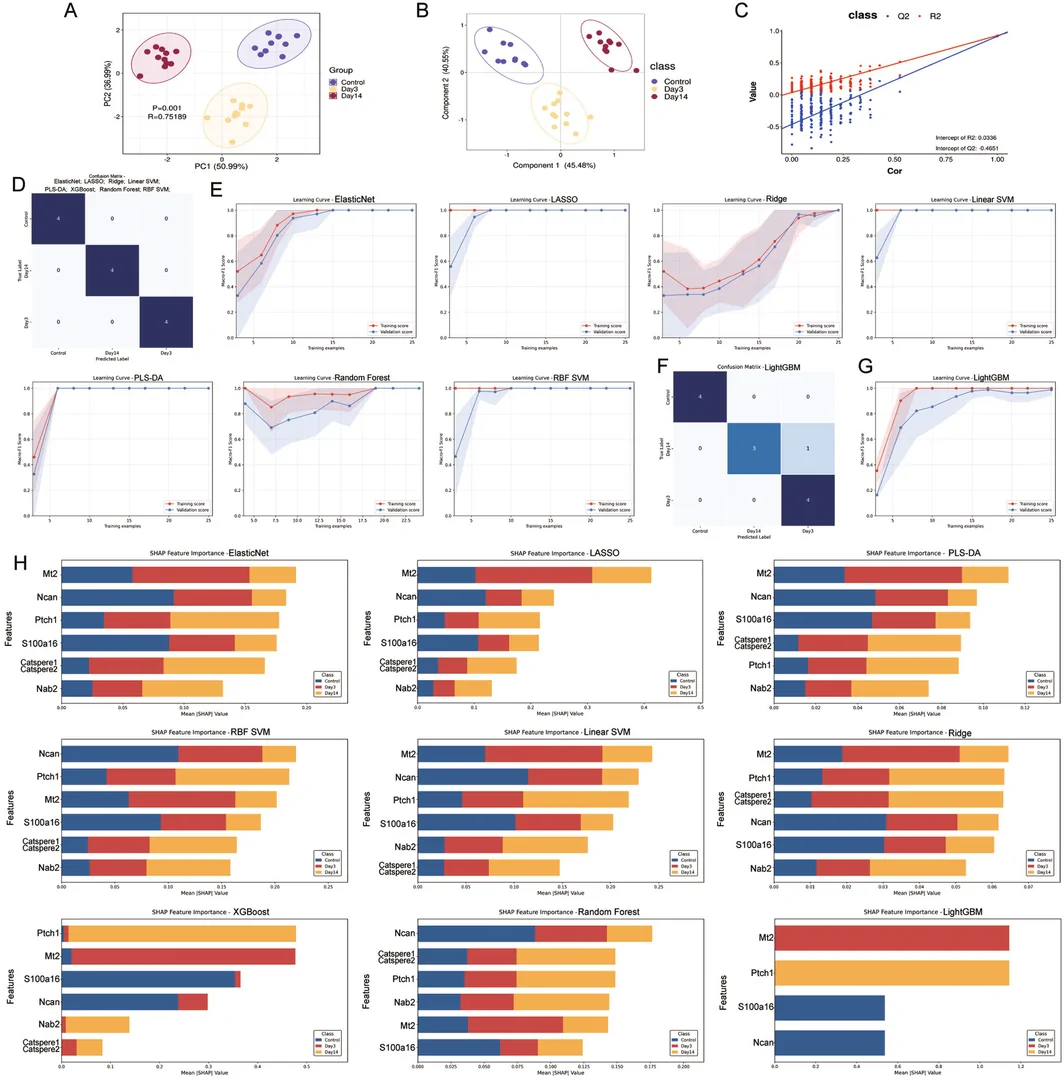
Performance evaluation of machine learning models based on the six‐gene mRNA signature. (A) PCA of the six core mRNA features. (B, C) PLS‐DA of the six core mRNA features. (B) Score plots. (C) Permutation test plots. (D) Confusion matrices of the ElasticNet, LASSO, Ridge, Linear SVM, PLS‐DA, XGBoost, RF, and RBF SVM models based on the six core mRNA features. (E) Learning curves of the ElasticNet, LASSO, Ridge, Linear SVM, PLS‐DA, RF, and RBF SVM models. (F, G) Confusion matrix (F) and learning curve (G) of the LightGBM model based on the six core mRNA features. (H) SHAP summary plots of the six core mRNA features across different machine learning models.

### 3.5 miRNA Features Robustly Distinguish Stress States but Show Limited Temporal Resolution

For miRNAs, RF and LASSO regression identified 44 and 12 candidate features, respectively. After integration with previously selected transcriptomic features, 217 candidate miRNAs were retained for model construction. However, three‐class classifiers based on this feature set failed to reliably distinguish the 3‐day and 14‐day stress groups, despite accurately separating stressed mice from controls (Figure S6A–I). These findings suggested that miRNAs were more suitable for identifying stress state than stress duration. Therefore, samples were reclassified into a binary framework (control vs. stress) for subsequent analyses.

Reanalysis identified 113 stress‐associated DEMs (Figure S6J). Integration with WGCNA (459 miRNAs, *r* = 0.537, *p* = 1.69 × 10^−4^) and machine learning feature selection yielded 104 candidate miRNAs for downstream modeling (Table S4). Further feature reduction using ElasticNet‐RFE and XGBoost identified a compact 11‐miRNA signature with strong discriminative performance (Figure 4A–H). Functional enrichment analysis indicated that these miRNAs were mainly associated with protein binding, metal ion binding, transcription factor activity, and nucleotide binding (Figure 4H).

**FIGURE 4:**
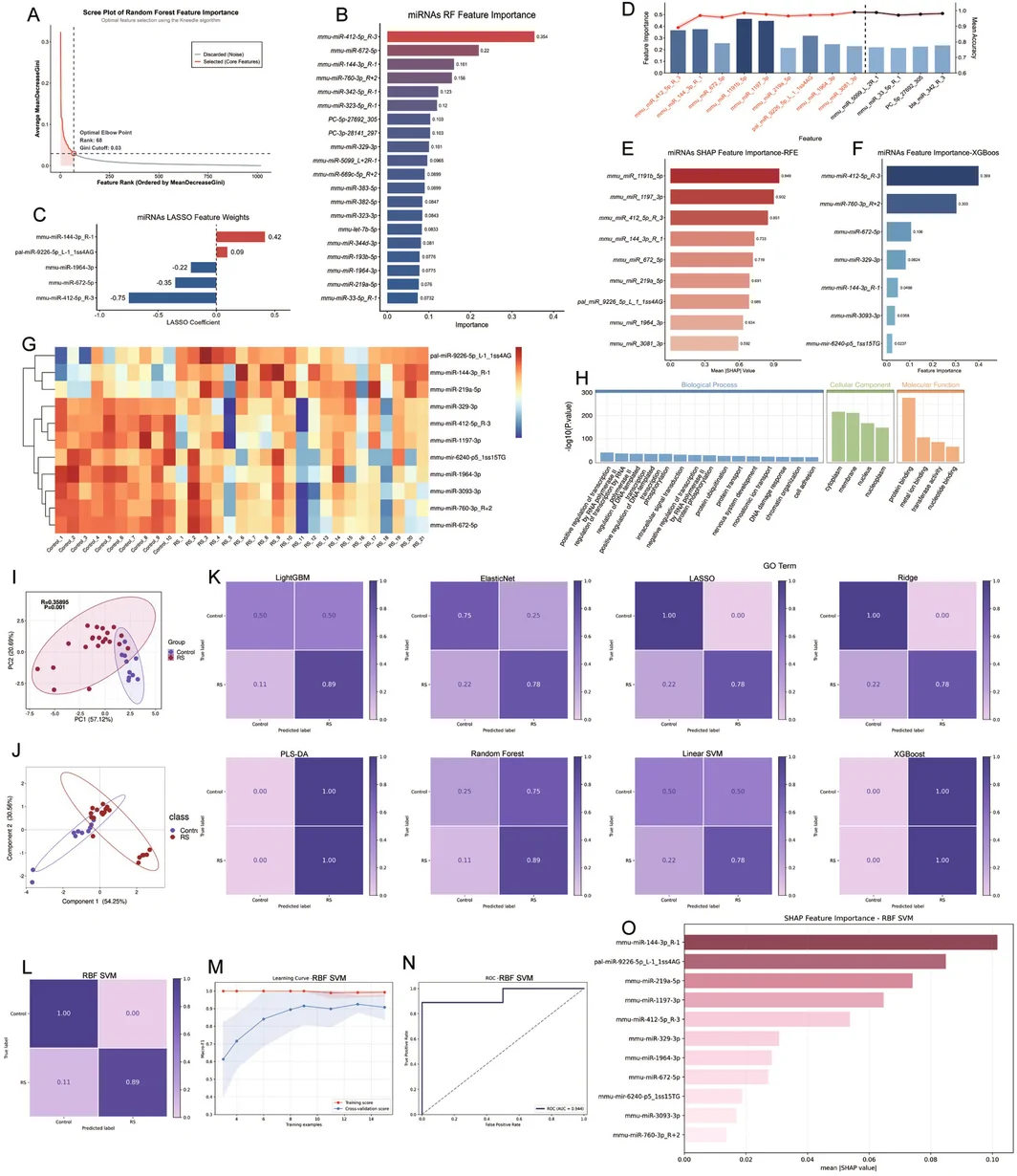
miRNA features robustly distinguish stress states but show limited temporal resolution. (A) Scree plot of feature importance derived from the RF model, with the mathematical elbow point identified using the Kneedle algorithm. (B) Top 20 miRNAs ranked by RF importance. (C) Five miRNAs selected by multinomial LASSO regression. (D) Feature reduction of the 104 miRNAs using the ElasticNet‐RFE strategy, showing optimal model performance with the top nine features. (E) SHAP summary plots of the nine miRNAs selected by ElasticNet‐RFE. (F) Seven feature miRNAs were initially selected by XGBoost, and six were retained after removing highly correlated features (Pearson's *r* > 0.95). (G) Heatmap showing the expression profiles of the 11 miRNA features used for model construction. (H) Functional enrichment of the 11 miRNA features used for model construction. (I) PCA of the 11 miRNA features. (J) PLS‐DA of the 11 miRNA features. (K) Confusion matrices of eight machine learning models based on the 11‐miRNA signature. (L–O) Confusion matrix (L), learning curve (M), ROC curve (N), and SHAP summary plot (O) of the RBF SVM model based on the 11‐miRNA signature.

Binary classification models reconstructed using the 11‐miRNA signature markedly outperformed the initial three‐class models. PCA (*r* = 0.359, *p* = 0.001) and PLS‐DA demonstrated clear separation between control and stress samples (Figure 4I,J). Among all evaluated algorithms, the RBF kernel SVM achieved the best performance in the held‐out test set, whereas Ridge, LASSO, and ElasticNet also showed robust classification ability (Figure 4K–N; Table S5). In contrast, RF, linear SVM, LightGBM, and XGBoost exhibited reduced generalizability or evidence of overfitting, indicating that nonlinear ensemble methods were less stable under the current sample size. SHAP analysis consistently identified mmu‐miR‐144‐3p, pal‐miR‐9226‐5p, and mmu‐miR‐219a‐5p as the major contributors to model prediction, with mmu‐miR‐144‐3p showing the highest overall contribution (Figure 4O). Collectively, these findings indicate that, unlike mRNAs, miRNAs have limited capacity for temporal stress stratification but provide robust molecular signatures for distinguishing stress status.

### 3.6 RT‐qPCR Validation of Core mRNA and miRNA

To validate the reproducibility and classification utility of the identified mRNA biomarkers, RT‐qPCR data were used to reconstruct machine‐learning models (Catspere1 was excluded because of poor validation performance: *p* > 0.05) (Figure 5A). Nested cross‐validation demonstrated robust predictive performance across multiple algorithms. Among them, Gaussian Naive Bayes achieved the best overall performance, whereas Shrinkage LDA, ExtraTrees, and both linear and RBF SVM also showed strong and consistent classification ability (Figure 5B–D; Table S6). Learning curves indicated stable model convergence with increasing training sample size, supporting good generalizability (Figure 5C; Figure S7). Although control samples were consistently classified with high accuracy, partial overlap remained between the 3‐ and 14‐day stress groups, suggesting that acute and chronic stress exhibit partially continuous molecular characteristics rather than a discrete biological transition. Feature importance analysis consistently identified Ncan as the dominant contributor across multiple models (Figure 5E), further supporting its role as a key biomarker for stress‐stage discrimination.

**FIGURE 5:**
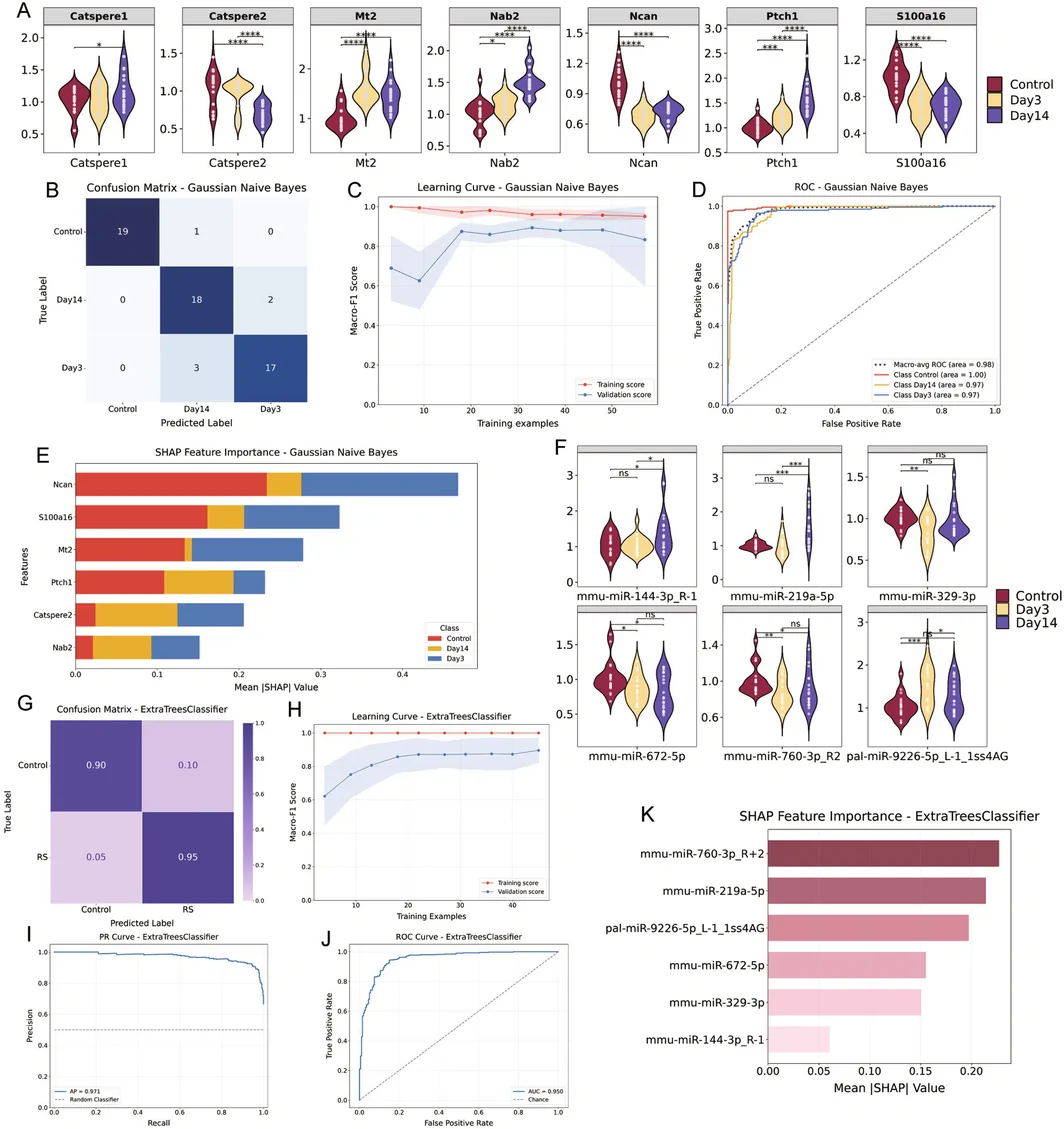
RT‐qPCR validation of the core mRNA and miRNA signatures. (A) RT‐qPCR validation of the expression levels of the six core mRNAs across different groups (*n* = 20). *p* < 0.05, **p < 0.01, and ****p < 0.0001 vs. the control. (B–E) Confusion matrix (B), learning curve (C), ROC curve (D), and SHAP summary plot (E) of the Gaussian Naive Bayes model constructed using RT‐qPCR expression data of the six core mRNAs. (F) RT‐qPCR validation of the expression levels of the six core miRNAs across different groups (*n* = 20). *p < 0.05, **p < 0.01, and ***p < 0.001 vs. the control. (G–K) Confusion matrix (G), learning curve (H), PR curve (I), ROC curve (J), and SHAP summary plot (K) of the ExtraTreesClassifier model constructed using RT‐qPCR expression data of the six core miRNAs.

For miRNAs, binary classification models were similarly constructed using RT‐qPCR data from six selected miRNAs (Figure 5F). ExtraTrees achieved the best classification performance, while Gaussian Naive Bayes and several other algorithms also demonstrated stable predictive ability (Figure 5G–J; Table S7). Learning curves confirmed good model convergence and generalization with increasing sample size (Figure S8). SHAP analysis consistently identified mmu‐miR‐672‐5p, mmu‐miR‐760‐3p_R + 2, and pal‐miR‐9226‐5p_L‐1_1ss4AG as the major contributors to stress‐state classification (Figure 5K). These RT‐qPCR validation results confirmed the robustness and reproducibility of both the mRNA‐ and miRNA‐based classifiers, supporting the translational potential of the identified molecular signatures for stress‐stage and stress‐state discrimination.

### 3.7 Ncan Is Preferentially Enriched in Astrocytes and Participates in Stress‐Associated PNNs Remodeling

To determine the cellular origin of the candidate genes, snRNA‐seq was used to characterize the cell‐type‐specific expression patterns of the seven machine‐learning–identified mRNAs. Ncan, Mt2, and Ptch1 were predominantly enriched in astrocytes, whereas Catspere2 was mainly expressed in GABAergic and glutamatergic neurons (Figure S9A). Among the astrocyte‐enriched genes, Ncan was consistently downregulated after both acute and chronic stress, whereas Mt2 and Ptch1 were upregulated (Figure S9B). None of these genes exhibited marked astrocyte subcluster specificity, suggesting that their expression changes reflect global astrocytic remodeling during stress (Figure 6A,B).

**FIGURE 6:**
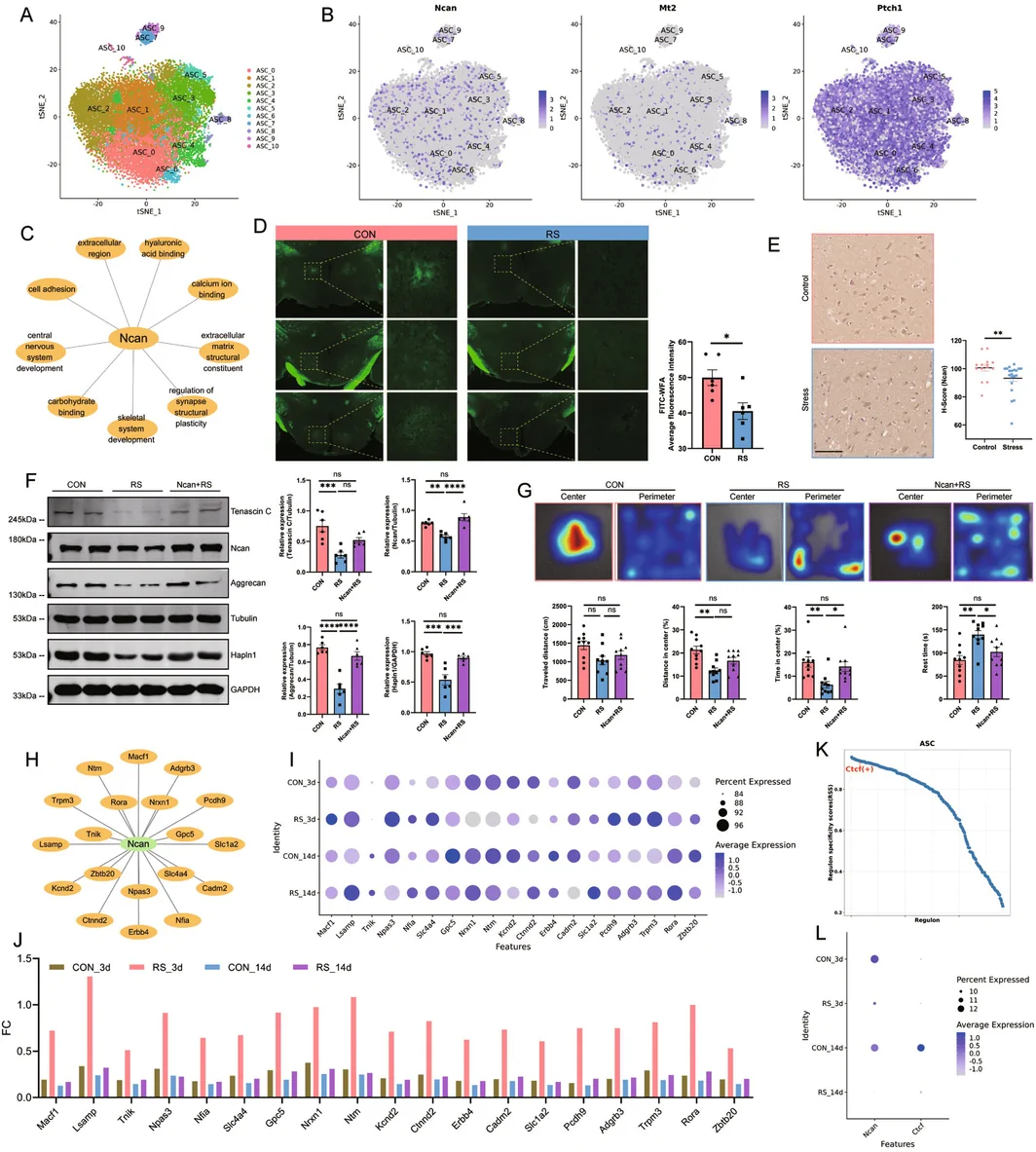
Ncan is preferentially enriched in astrocytes and participates in stress‐associated PNN remodeling. (A) t‐SNE plot showing astrocyte subpopulations identified from mouse hypothalamic snRNA‐seq. (B) Expression patterns of Ncan, Mt2, and Ptch1 across astrocyte subpopulations. (C) GO enrichment of Ncan‐associated biological processes based on snRNA‐seq. (D) Representative images and quantitative analysis of FITC‐WFA staining of PNNs in different hypothalamic regions of mice (*n* = 6). *p < 0.05 vs. the control. (E) Representative immunohistochemical staining and quantitative analysis of Ncan expression in human cortical tissues (*n* = 14 for controls and *n* = 20 for stress cases). **p < 0.01 vs. the control. (F) Representative Western blot images and densitometric quantification of Tenascin C, Aggrecan, Hapln1, and Ncan in the mouse hypothalamus (*n* = 6). **p < 0.01, ***p < 0.001, and ****p < 0.0001 vs. the control. (G) Open‐field test and tail suspension test results following recombinant Ncan treatment (*n* = 10). *p < 0.05 and **p < 0.01 vs. the control. (H) Intersection analysis of the top 30 perturbed genes identified by scTenifoldKnk virtual knockout in each group, yielding 19 shared regulatory genes. (I) Expression changes of the 19 shared regulatory genes across experimental groups based on snRNA‐seq data. (J) Fold‐change profiles of the 19 shared regulatory genes following Ncan virtual knockout across experimental groups. (K) Regulon specificity scores of Ctcf predicted by pySCENIC in astrocytes. (L) Expression patterns of Ctcf and Ncan across experimental groups based on snRNA‐seq data.

Given the consistent downregulation of Ncan across RNA‐seq, snRNA‐seq, and RT‐qPCR datasets, we further investigated its biological function. GO analysis associated Ncan with extracellular matrix organization, hyaluronic acid binding, and cell adhesion (Figure 6C). Consistent with its role as a core component of PNNs, stress markedly reduced hypothalamic PNN integrity (*p* = 0.0149, *F* = 1.115), accompanied by decreased Ncan expression (*p* = 0.0067, Mann–Whitney *U* = 64) (Figure 6D,E). Intracerebroventricular administration of recombinant Ncan protein restored the expression of major PNN components, including Tenascin C (*p* = 0.0006, *F* = 12.75), Aggrecan (*p* < 0.0001, *F* = 30.71), and Hapln1 (*p* = 0.0001, *F* = 17.76), and reduced stress‐induced depressive‐like behaviors in the open field (Time in center: *p* = 0.0012, Kruskal‐Wallis statistic = 13.52) and tail suspension tests (*p* = 0.0014, *F* = 8.490) (Figure 6F,G), supporting a protective role of Ncan in maintaining PNN homeostasis during stress.

To further explore the regulatory network of Ncan, scTenifoldKnk was performed for in silico gene perturbation analysis. Stress markedly increased the number of genes affected by Ncan knockout, particularly during acute stress, indicating enhanced network vulnerability under this condition (Table S8). Shared regulatory genes were primarily enriched in synaptic organization and neuronal projection–related processes (Figure 6H–J; Figure S9C,D), suggesting that Ncan may influence stress‐induced neural remodeling through coordinated regulation of synaptic function. Upstream regulatory analysis using pySCENIC identified Ctcf as a potential transcriptional regulator of Ncan, supported by highly concordant expression patterns and multiple predicted binding sites within the Ncan promoter (Figure 6K,L; Figure S9E,F). These findings identify astrocyte‐derived Ncan as a robust molecular marker of stress progression and suggest that its downregulation contributes to stress‐induced neural injury through disruption of extracellular matrix organization and PNN homeostasis.

## 4 Discussion

Stress‐related injury represents a major challenge in forensic medicine, neurobiology, and psychiatric research [25, 26, 27]. However, objective biomarkers with both temporal resolution and molecular stability remain lacking. Previous studies have primarily focused on inflammatory factors, endocrine hormones, or behavioral phenotypes, whereas the dynamic molecular remodeling underlying stress progression remains incompletely understood [27, 28, 29, 30]. A recent transcriptomics‐based workflow for cell‐type‐specific target discovery and therapeutic validation provided us with a research framework [31]. Moreover, most transcriptomic studies have focused on the hippocampus and prefrontal cortex [10, 11], while molecular alterations in the hypothalamus remain less well characterized. In the present study, we integrated whole‐transcriptome sequencing, snRNA‐seq, and multi‐layer machine‐learning analyses to establish a molecular framework for stress‐stage stratification and identify Ncan as a key regulator of hypothalamic stress responses. Our findings demonstrate that mRNAs and miRNAs exhibit distinct functional roles during stress progression and identify astrocyte‐derived Ncan as a potential regulator of stress‐induced neural injury.

A major finding of this study is the functional divergence between mRNAs and miRNAs. mRNAs showed superior ability to distinguish stress duration, whereas miRNAs more accurately reflected stress status. These differences are consistent with their biological functions: mRNAs directly reflect transcriptional activity and therefore respond rapidly to changes in stress intensity and duration [32, 33], whereas miRNAs primarily function as post‐transcriptional regulators that buffer transcriptional fluctuations and maintain network stability [34, 35]. Consistent with previous studies, mRNAs exhibited stage‐specific pathway alterations, shifting from lipid metabolism and inflammatory responses during early stress to steroid hormone biosynthesis and retinol metabolism during prolonged stress, suggesting progressive neuroendocrine dysregulation associated with sustained HPA‐axis activation [36, 37]. Lipid metabolic alterations are known to influence glial activation, neuroinflammation, and synaptic stability [38, 39, 40], while dysregulation of the endocannabinoid system has been closely linked to anxiety, depression, and HPA‐axis dysfunction [41, 42]. In contrast, miRNA‐associated pathways remained highly conserved across stress stages, mainly involving MAPK, Wnt, and axon‐guidance signaling, supporting a more stable role in stress adaptation and maintenance of transcriptional homeostasis.

A major strength of this study is the integration of transcriptomic analyses with machine‐learning approaches. Rather than relying solely on differential expression, we combined temporal clustering, co‐expression network analysis, and multiple machine‐learning algorithms to identify robust potential biomarkers. A compact six‐gene signature accurately classified stress stages, whereas an 11‐miRNA signature effectively distinguished stressed from control animals. RT‐qPCR validation further confirmed the reproducibility and generalizability of both classifiers. Interestingly, nonlinear models, including XGBoost and LightGBM, exhibited greater performance variability than linear models, suggesting that stress‐associated transcriptomic alterations may follow relatively stable low‐dimensional structures, consistent with previous studies of psychiatric disorders [43, 44]. Together, these findings indicate complementary roles for mRNAs and miRNAs, with mRNAs capturing temporal progression and miRNAs preferentially reflecting stress state. Consistent with previous reports linking miR‐144 and miR‐219 to neuroinflammation, myelination, and affective disorders [45, 46], mmu‐miR‐144‐3p showed the highest contribution to stress‐state classification.

Among all candidate biomarkers, Ncan exhibited the most stable expression pattern across RNA‐seq, snRNA‐seq, and RT‐qPCR datasets and consistently ranked among the most important features in multiple machine‐learning models. Ncan, a major chondroitin sulfate proteoglycan within the extracellular matrix and PNNs, plays an essential role in synaptic stability, neuronal plasticity, and circuit integrity [20, 21]. Consistent with previous reports of stress‐induced PNN disruption [21, 47, 48], we observed marked reductions in hypothalamic PNN integrity accompanied by sustained Ncan downregulation. Importantly, exogenous Ncan supplementation restored major PNN components and alleviated depressive‐like behaviors, suggesting that Ncan protects against stress‐induced neural injury by maintaining extracellular matrix and PNN homeostasis.

Our snRNA‐seq data further demonstrated that Ncan is predominantly expressed in astrocytes. Although stress research has traditionally emphasized neuronal dysfunction, astrocytes are increasingly recognized as key regulators of glutamate uptake [49, 50, 51], ionic homeostasis [39, 52], energy metabolism [53, 54], and extracellular matrix remodeling [55, 56]. Chronic stress has also been shown to induce astrocytic atrophy and reduced GFAP expression, thereby impairing neuronal support [57, 58]. Our findings suggest that stress‐induced reduction of astrocytic Ncan may weaken PNN stability, disrupt synaptic connectivity, and ultimately impair neural network homeostasis.

Network analyses provided additional mechanistic insight into Ncan regulation. scTenifoldKnk revealed markedly increased perturbation of the Ncan regulatory network under stress, particularly during the acute phase, indicating enhanced network vulnerability. The affected genes were mainly enriched in synapse maturation, cell projection organization, and membrane protein localization, suggesting that Ncan influences stress‐induced neural remodeling beyond extracellular matrix maintenance. Furthermore, pySCENIC identified Ctcf as a potential upstream transcriptional regulator of Ncan. Ctcf is a well‐known chromatin architecture regulator involved in neurodevelopment and stress‐related epigenetic regulation [59, 60]. Given the concordant expression patterns of Ctcf and Ncan, together with multiple predicted Ctcf binding sites within the Ncan promoter, our findings suggest a previously unrecognized Ctcf–Ncan regulatory axis involved in astrocyte‐mediated PNN homeostasis, although the underlying mechanisms require further experimental validation.

Several limitations should be acknowledged. First, these machine learning models were built on a relatively small mouse cohort and cannot be directly applied to the diagnosis or intervention of human diseases; thus, they require validation in larger independent datasets and clinical populations. Second, this study focused primarily on the hypothalamus, whereas stress‐related pathology involves coordinated alterations across multiple brain regions. Finally, although Ncan was identified as a key regulator of stress‐induced neural injury, the downstream molecular mechanisms linking astrocytic Ncan to PNN remodeling remain to be further elucidated.

## 5 Conclusion

This study systematically characterized hypothalamic transcriptomic remodeling during stress progression and revealed distinct roles of mRNAs and miRNAs in stress‐stage and stress‐state discrimination. We established robust transcriptome‐based classifiers and identified Ncan as the most informative biomarker across independent machine‐learning models. Astrocytic Ncan was consistently downregulated during stress, and its supplementation restored perineuronal net integrity and alleviated depressive‐like behaviors. These findings provide new insights into the molecular mechanisms underlying stress progression and suggest that Ncan may serve as a promising biomarker and a potential candidate for therapeutic intervention in stress‐related neural injury.

## Author Contributions

All authors contributed to the study design, implementation, and data collection. Weihao Zhu performed the experiments and drafted the manuscript. Chenyu Li, Mengting He, and Rui Shi analyzed the data. Xia Liu interpreted the results. Weibo Shi and Bin Cong designed the study. All authors contributed to the decision to submit the results for publication.

## Funding

This work was supported with funds from the National Natural Science Foundation of China (82293651, 82130055), the Hebei Province Innovation Capability Enhancement Plan Project (253A5601D), the Hebei Medical University Postdoctoral Fund (30705010083), and the Sanming Project of Medicine in Shenzhen (SZSM202411003).

## Ethics Statement

Animal studies were approved by the Laboratory Animal Ethical and Welfare Committee of Hebei Medical University (IACUC‐Hebmu‐2,023,011). Human brain tissue was approved by the Medical Ethics Committee of Hebei Medical University (P2022107).

## Conflicts of Interest

The authors declare no conflicts of interest.

## Supporting information


**Figure S1:** Quality assessment of transcriptomic sequencing data. (A, B) Quality control metrics of long RNA sequencing (A) and small RNA sequencing (B). (C, D) PCA based on mRNA, lncRNA, and circRNA expression profiles (C) and miRNA expression profiles (D).

**Figure S2:** Discriminative performance of different RNA types for stress conditions. (A) Three‐dimensional PCA based on mRNA, lncRNA, circRNA, and miRNA expression profiles. (B, C) PLS‐DA based on mRNA, lncRNA, circRNA, and miRNA expression profiles. (B) Score plots. (C) Permutation test plots.

**Figure S3:** Identification of common stress‐associated genes and miRNAs by integrating differential expression analysis and Mfuzz temporal clustering. (A, B) Overlap between DEGs and Mfuzz clusters, and the corresponding expression heatmap for mRNAs. (C, D) Overlap between DEMs and Mfuzz clusters, and the corresponding expression heatmap for miRNAs.

**Figure S4:** WGCNA of mRNA and miRNA expression profiles. (A) Soft‐thresholding analysis for mRNA. (B) Gene cluster dendrogram and module assignment for mRNA. (C) Module–trait association heatmap for mRNA. (D, E) GO enrichment of the MEbrown (D) and MElightcyan (E) modules. (F) Soft‐thresholding analysis for miRNA. (G) Gene cluster dendrogram and module assignment for miRNA. (H) Module–trait association heatmap for miRNA. (I, J) GO enrichment of the MEbrown (I) and MEblue (J) modules.

**Figure S5:** Performance of nine machine learning models constructed using the 304 candidate mRNA features. Confusion matrices (left) and learning curves (right) of (A) ElasticNet logistic regression; (B) LASSO logistic regression; (C) Ridge logistic regression; (D) Linear SVM; (E) LightGBM; (F) PLS‐DA; (G) RF; (H) RBF SVM; and (I) XGBoost.

**Figure S6:** Performance of multiclass machine learning models based on the 217 miRNA features. Confusion matrices (left) and learning curves (right) of (A) LASSO logistic regression; (B) Ridge logistic regression; (C) Linear SVM; (D) LightGBM; (E) PLS‐DA; (F) RF; (G) RBF SVM; (H) XGBoost; and (I) ElasticNet logistic regression. (J) Volcano plot of differential expression analysis for binary stress‐state classification based on the miRNA transcriptome.

**Figure S7:** Performance of machine learning models based on RT‐qPCR validation of the six core mRNA features using a nested cross‐validation framework. Confusion matrices (left) and learning curves (right) of (A) Dummy Classifier; (B) ElasticNet logistic regression; (C) Extra Trees classifier; (D) LASSO logistic regression; (E) Linear SVM; (F) PLS‐DA; (G) RBF SVM; (H) Ridge logistic regression; and (I) Shrinkage LDA.

**Figure S8:** Performance of machine learning models based on RT‐qPCR validation of the six core miRNA features using a nested cross‐validation framework. Confusion matrices (left) and learning curves (right) of (A) Dummy Classifier; (B) ElasticNet logistic regression; (C) Gaussian Naive Bayes; (D) LASSO logistic regression; (E) Linear SVM; (F) PLS‐DA; (G) RBF SVM; (H) Ridge logistic regression; and (I) Shrinkage LDA.

**Figure S9:** Additional evidence supporting the astrocyte‐specific expression and regulatory role of Ncan during stress‐associated PNNs remodeling. (A) Cell type‐specific expression of the six core mRNAs across major hypothalamic cell populations. (B) Differential expression of Ncan, Mt2, and Ptch1 in astrocytes across experimental groups. (C) Venn diagram showing the overlap among the top 30 perturbed genes identified by scTenifoldKnk virtual knockout in each experimental group. (D) GO enrichment of the 19 shared regulatory genes. (E) Area under the curve distribution of Ctcf regulon activity predicted by pySCENIC in astrocytes. (F) Predicted high‐confidence Ctcf binding sites within the Ncan promoter based on the JASPAR 2026 database.

**Table S1:** Results of screening meaningful biomarkers based on mRMA expression profile using various methods.

**Table S2:** Machine learning modeling results of 304 candidate genes.

**Table S3:** Machine learning modeling results of 6 candidate genes.

**Table S4:** Results of screening meaningful biomarkers based on miRMA expression profile using various methods.

**Table S5:** Machine learning modeling results of 11 miRNA.

**Table S6:** Machine learning modeling results of 6 genes (RT‐qPCR).

**Table S7:** Machine learning modeling results of 6 miRNA (RT‐qPCR).

**Table S8:** Results of ScTenifoldKnk virtual knockout of Ncan based on astrocyte expression profile.

## Data Availability

The whole‐transcriptome sequencing data generated in this study have been deposited in the GEO database under accession number [GSE346591 and GSE346592]. Other original contributions have been included in the main text or Supporting Information. Further inquiries can be directed to the corresponding author.

## References

[1] J. Ma , R. Wang , Y. Chen , Z. Wang , and Y. Dong , “5‐Ht Attenuates Chronic Stress‐Induced Cognitive Impairment in Mice Through Intestinal Flora Disruption,” Journal of Neuroinflammation 201 (2023): 23. 10.1186/s12974-023-02693-1 PMC9896737 36737776

[2] J. García‐Cancela , O. Conde‐Vázquez , S. Navarro‐Ledesma , and L. Pruimboom , “Neuroinflammation, Metabolic Dysfunction, and Chronic Stress Converge on the Insular Cortex in Chronic Pain,” Neuroscience and Biobehavioral Reviews 188 (2026): 106791. 10.1016/j.neubiorev.2026.106791 42235664

[3] E. Knezevic , K. Nenic , V. Milanovic , and N. N. Knezevic , “The Role of Cortisol in Chronic Stress, Neurodegenerative Diseases, and Psychological Disorders,” Cells 1223 (2023): 2726. 10.3390/cells12232726 PMC10706127 38067154

[4] Y. Cao , Y. Zhao , T. Deng , et al., “Hepatic Acetyl‐Coa Metabolism Modulates Neuroinflammation and Depression Susceptibility via Acetate,” Cell Metabolism 3711 (2025): 2185–2201.e8. 10.1016/j.cmet.2025.08.010 40992374

[5] X. Ma , W. Shi , Z. Wang , et al., “Butyric Acid and Valeric Acid Attenuate Stress‐Induced Ferroptosis and Depressive‐Like Behaviors by Suppressing Hippocampal Neuroinflammation,” Journal of Translational Medicine 231 (2025): 974. 10.1186/s12967-025-06950-0 PMC12403447 40898211

[6] K. Chen , B. Hollunder , M. Garbusow , M. Sebold , and A. Heinz , “The Physiological Responses to Acute Stress in Alcohol‐Dependent Patients: A Systematic Review,” European Neuropsychopharmacology: The Journal of the European College of Neuropsychopharmacology 41 (2020): 1–15. 10.1016/j.euroneuro.2020.09.003 32994116

[7] J. Vyverman , R. De Baere , I. Timmers , I. Coppieters , J. Van Oosterwijck , and M. Moerkerke , “The Stress‐Pain Connection in Chronic Primary Pain: A Systematic Review and Meta‐Analysis of Physiological Stress Markers in Relation to Experimental Pain Responses,” Neuroscience and Biobehavioral Reviews 184 (2026): 106604. 10.1016/j.neubiorev.2026.106604 41679412

[8] H. Zhou , X. Feng , J. Wen , et al., “A Skin‐Hypothalamus Axis Couples Heat Stress and Metabolic Dysfunction,” Cell 18912 (2026): 3571–3588. 10.1016/j.cell.2026.03.045 42019490

[9] S. Hu , J. Huang , Q. Zhang , et al., “Acute and Chronic Stress Differentially Regulate Pain via Distinct Ensembles in the Paraventricular Nucleus of Hypothalamus,” Molecular Psychiatry 312 (2026): 649–663. 10.1038/s41380-025-03144-4 40781546

[10] G. S. S. Tofani , S. Leigh , C. E. Gheorghe , et al., “Gut Microbiota Regulates Stress Responsivity via the Circadian System,” Cell Metabolism 371 (2025): 138–153. 10.1016/j.cmet.2024.10.003 39504963

[11] A. Floriou‐Servou , L. von Ziegler , L. Stalder , et al., “Distinct Proteomic, Transcriptomic, and Epigenetic Stress Responses in Dorsal and Ventral Hippocampus,” Biological Psychiatry 847 (2018): 531–541. 10.1016/j.biopsych.2018.02.003 29605177

[12] K. Paunovska , D. Loughrey , and J. E. Dahlman , “Drug Delivery Systems for Rna Therapeutics,” Nature Reviews. Genetics 235 (2022): 265–280. 10.1038/s41576-021-00439-4 PMC8724758 34983972

[13] Y. Huang , S. Zhu , S. Yao , H. Zhai , C. Liu , and J. J. Han , “Unraveling Aging From Transcriptomics,” Trends in Genetics: TIG 413 (2025): 218–235. 10.1016/j.tig.2024.09.006 39424502

[14] B. Chen , X. Sun , H. Huang , C. Feng , W. Chen , and D. Wu , “An Integrated Machine Learning Framework for Developing and Validating a Diagnostic Model of Major Depressive Disorder Based on Interstitial Cystitis‐Related Genes,” Journal of Affective Disorders 359 (2024): 22–32. 10.1016/j.jad.2024.05.061 38754597

[15] C. Hu , H. Li , J. Cui , et al., “Integrative Analysis Identifies Il‐6/Jun/Mmp‐9 Pathway Destroyed Blood‐Brain‐Barrier in Autism Mice via Machine Learning and Bioinformatic Analysis,” Translational Psychiatry 151 (2025): 239. 10.1038/s41398-025-03452-x PMC12254206 40645949

[16] C. J. Huseby , E. Delvaux , D. L. Brokaw , and P. D. Coleman , “Blood Transcript Biomarkers Selected by Machine Learning Algorithm Classify Neurodegenerative Diseases Including Alzheimer's Disease,” Biomolecules 1211 (2022): 1592. 10.3390/biom12111592 PMC9687215 36358942

[17] J. W. Fawcett , T. Oohashi , and T. Pizzorusso , “The Roles of Perineuronal Nets and the Perinodal Extracellular Matrix in Neuronal Function,” Nature Reviews. Neuroscience 208 (2019): 451–465. 10.1038/s41583-019-0196-3 31263252

[18] J. W. Fawcett , M. Fyhn , P. Jendelova , J. C. F. Kwok , J. Ruzicka , and B. A. Sorg , “The Extracellular Matrix and Perineuronal Nets in Memory,” Molecular Psychiatry 278 (2022): 3192–3203. 10.1038/s41380-022-01634-3 PMC9708575 35760878

[19] S. Tansley , N. Gu , A. U. Guzmán , et al., “Microglia‐Mediated Degradation of Perineuronal Nets Promotes Pain,” Science (New York, N.Y.) 377, no. 6601 (2022): 80–86. 10.1126/science.abl6773 35617374

[20] S. Spijker , M. Koskinen , and D. Riga , “Incubation of Depression: Ecm Assembly and Parvalbumin Interneurons After Stress,” Neuroscience and Biobehavioral Reviews 118 (2020): 65–79. 10.1016/j.neubiorev.2020.07.015 32687884

[21] Y. Mei , H. Chen , Q. Cao , et al., “Semaphorin 3a‐Mediated Perineuronal Nets Formation Incubates Depressive‐Like Behaviors in Male Mice via Activating Parvalbumin‐Expressing Interneurons,” Molecular Psychiatry 313 (2026): 1276–1292. 10.1038/s41380-025-03239-y 40926092

[22] M. Rincón‐Cortés , J. P. Herman , S. Lupien , J. Maguire , and R. M. Shansky , “Stress: Influence of Sex, Reproductive Status and Gender,” Neurobiology of Stress 10 (2019): 100155. 10.1016/j.ynstr.2019.100155 PMC6430637 30949564

[23] F. Homo‐Delarche , F. Fitzpatrick , N. Christeff , E. A. Nunez , J. F. Bach , and M. Dardenne , “Sex Steroids, Glucocorticoids, Stress and Autoimmunity,” Journal of Steroid Biochemistry and Molecular Biology 404‐6 (1991): 619–637. 10.1016/0960-0760(91)90285-d 1958562

[24] L. Steuernagel , B. Y. H. Lam , P. Klemm , et al., “Hypomap‐a Unified Single‐Cell Gene Expression Atlas of the Murine Hypothalamus,” Nature Metabolism 410 (2022): 1402–1419. 10.1038/s42255-022-00657-y PMC9584816 36266547

[25] B. S. McEwen , “Physiology and Neurobiology of Stress and Adaptation: Central Role of the Brain,” Physiological Reviews 873 (2007): 873–904. 10.1152/physrev.00041.2006 17615391

[26] L. Oprinca‐Muja , C. Mohor , G. Oprinca , et al., “Burnout Syndrome in Forensic Medicine and Its Association With Vicarious Trauma, Posttraumatic Stress Syndrome and Occupational Stress,” International Journal of Legal Medicine 1393 (2025): 1223–1237. 10.1007/s00414-024-03398-7 PMC12003476 39792327

[27] X. Feng , M. Jia , M. Cai , T. Zhu , K. Hashimoto , and J. Yang , “Central‐Peripheral Neuroimmune Dynamics in Psychological Stress and Depression: Insights From Current Research,” Molecular Psychiatry 3010 (2025): 4881–4898. 10.1038/s41380-025-03085-y PMC12436188 40610703

[28] K. P. J. Wright , A. L. Drake , D. J. Frey , et al., “Influence of Sleep Deprivation and Circadian Misalignment on Cortisol, Inflammatory Markers, and Cytokine Balance,” Brain, Behavior, and Immunity 47 (2015): 24–34. 10.1016/j.bbi.2015.01.004 PMC5401766 25640603

[29] A. Vezzani , S. Balosso , and T. Ravizza , “Neuroinflammatory Pathways as Treatment Targets and Biomarkers in Epilepsy,” Nature Reviews. Neurology 158 (2019): 459–472. 10.1038/s41582-019-0217-x 31263255

[30] S. Cohen , D. Janicki‐Deverts , W. J. Doyle , et al., “Chronic Stress, Glucocorticoid Receptor Resistance, Inflammation, and Disease Risk,” Proceedings of the National Academy of Sciences of the United States of America 109, no. 16 (2012): 5995–5999. 10.1073/pnas.1118355109 PMC3341031 22474371

[31] D. Lin , Y. Huang , S. Wu , et al., “Precise Nanodelivery of Screened Compounds Alleviates Sepsis‐Associated Encephalopathy by Targeting Microglial anxa2,” Science Bulletin 71, no. 16 (2026): 4244–4271. 10.1016/j.scib.2026.07.059 42562700

[32] M. Stewart , “From Transcription to Export: Mrna's Winding Path to the Cytoplasm,” Trends in Biochemical Sciences 50, no. 9 (2025): 748–765. 10.1016/j.tibs.2025.06.004 PMC7619002 40670258

[33] S. Jana , S. Brahma , S. Arora , et al., “Transcriptional‐Translational Conflict Is a Barrier to Cellular Transformation and Cancer Progression,” Cancer Cell 41, no. 5 (2023): 853–870. 10.1016/j.ccell.2023.03.021 PMC10208629 37084735

[34] M. Ha and V. N. Kim , “Regulation of Microrna Biogenesis,” Nature Reviews. Molecular Cell Biology 15, no. 8 (2014): 509–524. 10.1038/nrm3838 25027649

[35] S. Li , C. Castillo‐González , B. Yu , and X. Zhang , “The Functions of Plant Small Rnas in Development and in Stress Responses,” Plant journal: for cell and molecular biology 90, no. 4 (2017): 654–670. 10.1111/tpj.13444 27943457

[36] M. S. Oitzl , D. L. Champagne , R. van der Veen , and E. R. de Kloet , “Brain Development Under Stress: Hypotheses of Glucocorticoid Actions Revisited,” Neuroscience and Biobehavioral Reviews 346 (2010): 853–866. 10.1016/j.neubiorev.2009.07.006 19631685

[37] S. G. Nunez , S. P. Rabelo , N. Subotic , J. W. Caruso , and N. N. Knezevic , “Chronic Stress and Autoimmunity: The Role of Hpa Axis and Cortisol Dysregulation,” International Journal of Molecular Sciences 2620 (2025): 9994. 10.3390/ijms26209994 PMC12563903 41155288

[38] Y. Mi , G. Qi , F. Vitali , et al., “Loss of Fatty Acid Degradation by Astrocytic Mitochondria Triggers Neuroinflammation and Neurodegeneration,” Nature Metabolism 53 (2023): 445–465. 10.1038/s42255-023-00756-4 PMC10202034 36959514

[39] D. Wu , Y. Ma , B. Wang , et al., “Pathological Roles of Astrocytes in Traumatic Brain Injury,” CNS Neuroscience & Therapeutics 324 (2026): 28. 10.1002/cns.70856 PMC13070886 41968392

[40] Y. Cheng , Y. Zhou , Y. Chen , et al., “Cholesterol Drives ifitm3+Microglia Activation and Induces Sting Mediated Neuroinflammation After Ischemic Stroke,” CNS Neuroscience & Therapeutics 32, no. 8 (2026): 16. 10.1002/cns.71073 PMC13459068 42578421

[41] V. Micale and F. Drago , “Endocannabinoid System, Stress and Hpa Axis,” European Journal of Pharmacology 834 (2018): 230–239. 10.1016/j.ejphar.2018.07.039 30036537

[42] R. Mechoulam and L. A. Parker , “The Endocannabinoid System and the Brain,” Annual Review of Psychology 64, no. 64 (2013): 21–47. 10.1146/annurev-psych-113011-143739 22804774

[43] T. Tuersong , Y. X. Yong , Y. Chen , et al., “Integrating Plasma Circulating Protein‐Centered Multi‐Omics to Identify Potential Therapeutic Targets for Parkinsonian Cognitive Disorders,” Journal of Translational Medicine 231 (2025): 535. 10.1186/s12967-025-06541-z PMC12070786 40355913

[44] B. Aguzzoli Heberle , K. L. Fox , L. Lobraico Libermann , et al., “Systematic Review and Meta‐Analysis of Bulk Rnaseq Studies in Human Alzheimer's Disease Brain Tissue,” Alzheimer's & Dementia: The Journal of the Alzheimer's Association 213 (2025): e70025. 10.1002/alz.70025 PMC11881636 40042520

[45] A. Yadegari , F. Tahmasebi , E. R. Asl , Z. Vahidinia , and S. Barati , “Dual‐Targeting Mir‐219 and Mir‐146a in Multiple Sclerosis: Bridging Myelin Repair and Neuroinflammation for Novel Therapeutic Insights,” Cellular and Molecular Neurobiology 461 (2026): 22. 10.1007/s10571-025-01661-y PMC12847516 41498857

[46] J. Zhao , Y. Zhou , S. Cheng , J. Shen , Y. Li , and Z. Xu , “Mir‐144 Regulates Cognitive Dysfunction via nlrp3 Inflammasome and foxo1/Adipor Pathway in t2dm Mice,” Molecular Neurobiology 631 (2026): 610. 10.1007/s12035-026-05897-7 42086968

[47] Y. Dong , K. Zhao , X. Qin , G. Du , and L. Gao , “The Mechanisms of Perineuronal Net Abnormalities in Contributing Aging and Neurological Diseases,” Ageing Research Reviews 92 (2023): 14. 10.1016/j.arr.2023.102092 37839757

[48] Z. A. Colon , S. C. Chan , and K. A. Maguire‐Zeiss , “Age‐Related Inflammatory Changes and Perineuronal Net Dynamics: Implications for Aging,” Journal of Neuroinflammation 221 (2025): 17. 10.1186/s12974-025-03568-3 PMC12628600 41254697

[49] S. Mahmoud , M. Gharagozloo , C. Simard , A. Amrani , and D. Gris , “Nlrx1 Enhances Glutamate Uptake and Inhibits Glutamate Release by Astrocytes,” Cells 85 (2019): 400. 10.3390/cells8050400 PMC6562695 31052241

[50] F. Birey , M. Kloc , M. Chavali , et al., “Genetic and Stress‐Induced Loss of ng2 Glia Triggers Emergence of Depressive‐Like Behaviors Through Reduced Secretion of fgf2,” Neuron 88 (2015): 941–956. 10.1016/j.neuron.2015.10.046 PMC5354631 26606998

[51] G. Sanacora and M. Banasr , “From Pathophysiology to Novel Antidepressant Drugs: Glial Contributions to the Pathology and Treatment of Mood Disorders,” Biological Psychiatry 73, no. 12 (2013): 1172–1179. 10.1016/j.biopsych.2013.03.032 PMC3688253 23726152

[52] A. Verkhratsky , L. M. B. Hansen , C. Staehr , and V. V. Matchkov , “The Astrocytic Engine: Na(+),k(+)‐Atpase at the Nexus of Brain Function and Malfunction,” American Journal of Physiology. Cell Physiology 330, no. 1 (2026): C238–C251. 10.1152/ajpcell.00814.2025 41364026

[53] M. Bélanger , I. Allaman , and P. J. Magistretti , “Brain Energy Metabolism: Focus on Astrocyte‐Neuron Metabolic Cooperation,” Cell Metabolism 14, no. 6 (2011): 724–738. 10.1016/j.cmet.2011.08.016 22152301

[54] Z. Chen , Z. Yuan , S. Yang , et al., “Brain Energy Metabolism: Astrocytes in Neurodegenerative Diseases,” CNS Neuroscience & Therapeutics 29, no. 1 (2023): 24–36. 10.1111/cns.13982 PMC9804080 36193573

[55] A. Sinha , A. M. Kowalchuk , N. Khatibi , R. T. Matthews , and J. L. MacDonald , “Astrocytic Regulation of Aberrant Perineuronal Net Formation in mecp2‐Null Neocortex,” Journal of Neurochemistry 170, no. 6 (2026): 18. 10.1111/jnc.70503 PMC13316734 42375118

[56] N. John , H. Krügel , R. Frischknecht , et al., “Brevican‐Containing Perineuronal Nets of Extracellular Matrix in Dissociated Hippocampal Primary Cultures,” Molecular and Cellular Neuroscience 314 (2006): 774–784. 10.1016/j.mcn.2006.01.011 16503162

[57] F. Guo , J. Fan , J. Liu , et al., “Astrocytic alkbh5 in Stress Response Contributes to Depressive‐Like Behaviors in Mice,” Nature Communications 151 (2024): 4347. 10.1038/s41467-024-48730-2 PMC11109195 38773146

[58] B. Zhou , Y. Zuo , and R. Jiang , “Astrocyte Morphology: Diversity, Plasticity, and Role in Neurological Diseases,” CNS Neuroscience & Therapeutics 256 (2019): 665–673. 10.1111/cns.13123 PMC6515705 30929313

[59] D. P. Lee , W. L. W. Tan , C. G. Anene‐Nzelu , et al., “Robust Ctcf‐Based Chromatin Architecture Underpins Epigenetic Changes in the Heart Failure Stress‐Gene Response,” Circulation 139, no. 16 (2019): 1937–1956. 10.1161/CIRCULATIONAHA.118.036726 30717603

[60] S. Chakraborty , N. Wenzlitschke , M. J. Anderson , et al., “Deletion of a Single Ctcf Motif at the Boundary of a Chromatin Domain With Three Fgf Genes Disrupts Gene Expression and Embryonic Development,” Developmental Cell 6013 (2025): 1838–1853. 10.1016/j.devcel.2025.02.002 PMC12237608 40015278

